# Evaluating DNA damage in a South American marsupial through exfoliated cells of the buccal mucosa

**DOI:** 10.1007/s11356-026-37406-7

**Published:** 2026-02-20

**Authors:** Hermes Willyan Parreira Claro, Daniela de Melo e Silva, Wellington Hannibal

**Affiliations:** 1 Laboratório de Mutagênese (LabMut), Instituto de Ciências Biológicas, Universidade Federal de Goiás, Campus Samambaia, Goiânia, Goiás 74690-900 Brazil; 2 Laboratório de Ecologia e Biogeografia de Mamíferos, Centro de Pesquisa em Biodiversidade e Seviços Ecossistêmicoss, Universidade Estadual de Goiás, Campus Sudoeste, Quirinópolis, Goiás 75862-196 Brazil

**Keywords:** Biomarker, Buccal mucosa, Conservation, Micronucleus test, Genotoxic damage, In situ study

## Abstract

The edge effect alters microclimatic conditions and increases heavy metal pollution in monoculture landscapes, thereby exposing wildlife to toxic substances. This highlights the need for genotoxicity assessments in fragmented environments. The micronucleus test is a valuable biomarker for detecting DNA damage, though its use in wild mammals is recent. In this pioneering study, we applied the text to exfoliated buccal mucosa cells of *Gracilinanus agilis*, a South American marsupial, using the micronucleus test. Individuals were captured in a semideciduous forest in southern Goiás, central Brazil. We collected buccal cells from 41 individuals (13 females and 28 males) across 13 sampling sites. We identified six types of nuclear abnormalities: micronuclei, karyolysis, pyknosis, binucleated cells, karyorrhexis, and nuclear buds. Karyolysis, pyknosis, and binucleated cells were most frequent at the forest edge compared to the interior, indicating greater genotoxic stress in edge habitats. Our findings support the use of the micronucleus test as a non-invasive tool for monitoring DNA damage in wild populations and demonstrate that marsupials inhabiting forest edges are more susceptible to genotoxic effects.

**Graphical Abstract:**

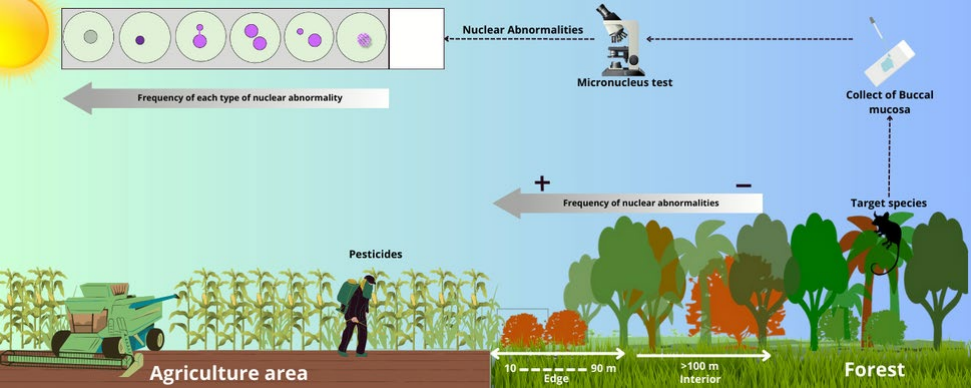

## Introduction

The edge effect exposes wildlife to altered abiotic and biotic conditions, including increased solar radiation, temperature fluctuations, and reduced humidity (Laurance et al. 2002). These microclimatic shifts can intensify physiological stress in wildlife and modulate exposure to contaminants that accumulate in soils and vegetation, especially in agricultural landscapes dominated by monocultures (Alengebawy et al. 2021). As many mammalian species persist in these disturbed environments (Gomes-de-Sá et al. 2025), there is growing concern regarding the sublethal effects of xenobiotics on their genomic integrity. Understanding how landscape degradation and agricultural intensification interact to influence genotoxicity is therefore essential for evaluating the health of wildlife population in fragmented landscapes.

The Cerrado biome, a major agricultural frontier in Brazil, has experienced extensive conversion to crop and pasturelands, resulting in widespread contamination risks associated with intensive use, especially in soybean, corn, and sugarcane cultivation (Pignati et al. 2017). In this scenario, the state of Goiás ranks among the highest consumers of agrochemical in the country (IBAMA 2024) . Given the proximity between remnant vegetation fragments and agricultural fields, wildlife species inhabiting forest edges may face elevated exposure to pesticide and other xenobiotics in the region.

Biomarkers capable of detecting early, subcellular responses to environmental stressors offer an effective approach to assessing the impact of contamination in wild mammals. Among these biomarkers, the micronucleus (MN) test, based on the analysis of exfoliated buccal mucosa cells, has been established as a biomarker for detecting DNA damage in humans resulting from exposure to genotoxic agents. The test relies on the identification of micronuclei formed when chromosome fragments or entire chromosomes fail to be incorporated into the daughter nuclei during mitosis, forming additional, smaller nuclei distinct from the main one (Ramirez and Saldanha 2002). Due to its non-invasive approach, low cost, and sensitivity (Thomas et al. 2009; Bolognesi et al. 2013), the MN test has been increasingly applied to ecotoxicological studies.

Given its effectiveness in detecting DNA damage in humans, the micronucleus test has been successfully adapted for use in a variety of wild and domestic mammal species, including bats (Benvindo-Souza et al. 2020), bovines (Ferré et al. 2024), dogs and cats (Santovito et al. 2022), and marsupials and rodents (Borges-Almeida et al. 2025). These studies demonstrate that chromosomal aberrations—such as fragments and lagging chromosomes— can be reliably detected across taxa, reinforcing the potential of the MN test for monitoring DNA damage in wild mammals. However, despite the ecological importance of Neotropical marsupials, their use in genotoxicity biomonitoring remains poorly explored (Borges-Almeida et al. 2025).

South American marsupials represent an important mammalian group for ecological studies due to their diversity in species, diet, body size, habitat use, and locomotor habits (Gardner 2008). Their intricate relationships with environmental quality gradients (Hannibal et al. 2020) and matrix use (Passamani and Ribeiro 2009; Santos-Filho et al. 2008) make them particularly susceptible to the accumulation of heavy metals, such as lead, copper, and cadmium (Costa et al. 2023). Yet toxicological studies in South America marsupials have been limited to the species *Monodelphis domestica* (Fadem et al. 1982) and *Marmosa murina* (Costa et al. 2023), with few applications in in situ research.

Recent evidence from the Cerrado indicates that fragmentation can mediate cytogenetic responses in marsupials. Individuals sampled at forest edges within agricultural landscapes exhibit higher frequencies of micronuclei and other nuclear abnormalities (Borges-Almeida et al. 2025; Costa et al. 2023), suggesting that landscape configuration interacts with agrochemical exposure to compromise genomic stability. These findings highlight the value of cytogenetic biomarkers for detecting sublethal effects in wildlife inhabiting fragmented agroecosystems.

Selecting a species with a broader geographic distribution and ecological adaptability is essential to enhance the scope and applicability of genotoxicity biomarkers in marsupials. The Agile Gracile Opossum, *Gracilinanus agilis* (Burmeister, 1854), is a small (12–41 g) South American marsupial belonging to the family Didelphidae. This species is associated with both open and forested environments (Antunes et al. 2021) and exhibits significant tolerance to anthropogenic disturbances, thriving in early-stage regenerating fragments. It feeds on arthropods, including Hymenoptera, Coleoptera, and Isoptera (Claro and Hannibal 2022), while also acting as a seed disperser and a predator of agricultural pests (Camargo et al. 2014, 2022). Given its adaptability and small size, *G. agilis* is an ideal marsupial model for ecotoxicology studies.

Despite the growing use of the micronucleus test to assess genotoxicity in mammals (Borges-Almeida et al. 2025; Benvindo-Souza et al. 2020; Ferré et al. 2024; Santovito et al. 2022), no studies to date have focused on *G. agilis*. To fill this gap, we applied the micronucleus test to exfoliated buccal mucosa cells of a South American marsupial. We hypothesized that individuals inhabiting forest edges would exhibit a higher frequency of mutagenic alterations—such as micronuclei and other nuclear abnormalities—compared to those living in the forest interior.

## Materials and methods

### Study area

**Fig. 1:**
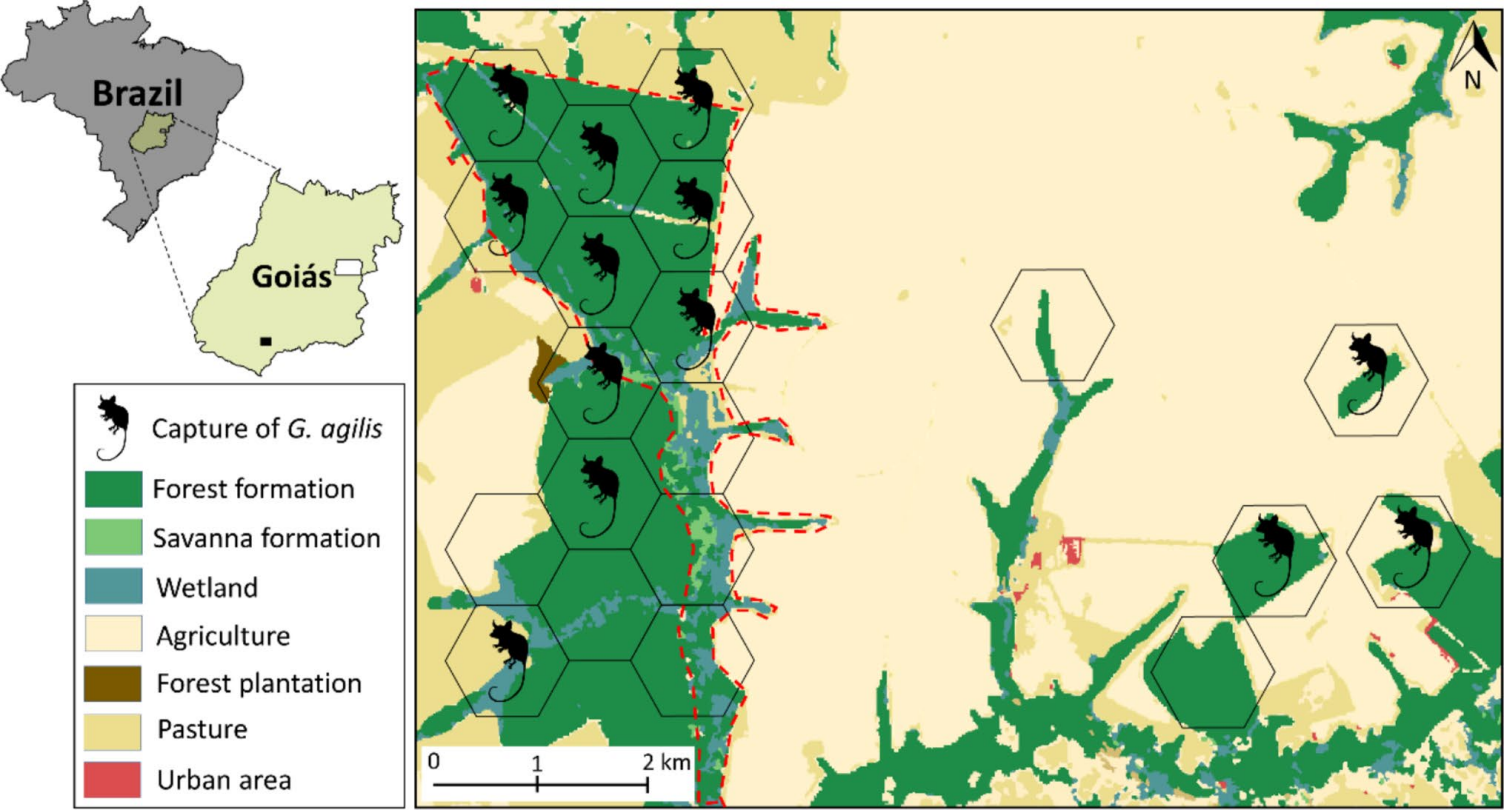
Map showing the Serra da Fortaleza Wildlife Refuge (dashed red) in a fragmented landscape in southern Goiás (black square), central Brazil. Hexagons represent the sampling units

The sampling was conducted in the protected area Serra da Fortaleza Wildlife Refuge (SFWR) situated at Quirinópolis municipality (18.2456°S, 050.6847°W) in southern Goiás, central Brazil. The SFWR is approximately 490 ha and composed of gallery forests, semideciduous forests, and shrub-land formations, which are surrounded by a fragmented landscape of sugar cane, soybean, corn, and pasture plantations (Fig. 1). We divided the fragmented landscape in 20 sampling units (hexagons of 57 ha each), distributed between the protected area and surroundings (Fig. 1).

The local climate is classified as Aw, with two well-defined seasons: rainy (October to March) and dry (April to September), according to the Köppen classification. Annual rainfall varies on average from 1500 to 1750 mm. The average annual temperature has little seasonal variation, averaging 23.8 °C, with the highest values in October, at 24.5 °C, and the lowest values in July, at 20.8 °C (Alvares et al. 2014).

### Capture of* Gracilinanus agilis*

**Fig. 2:**
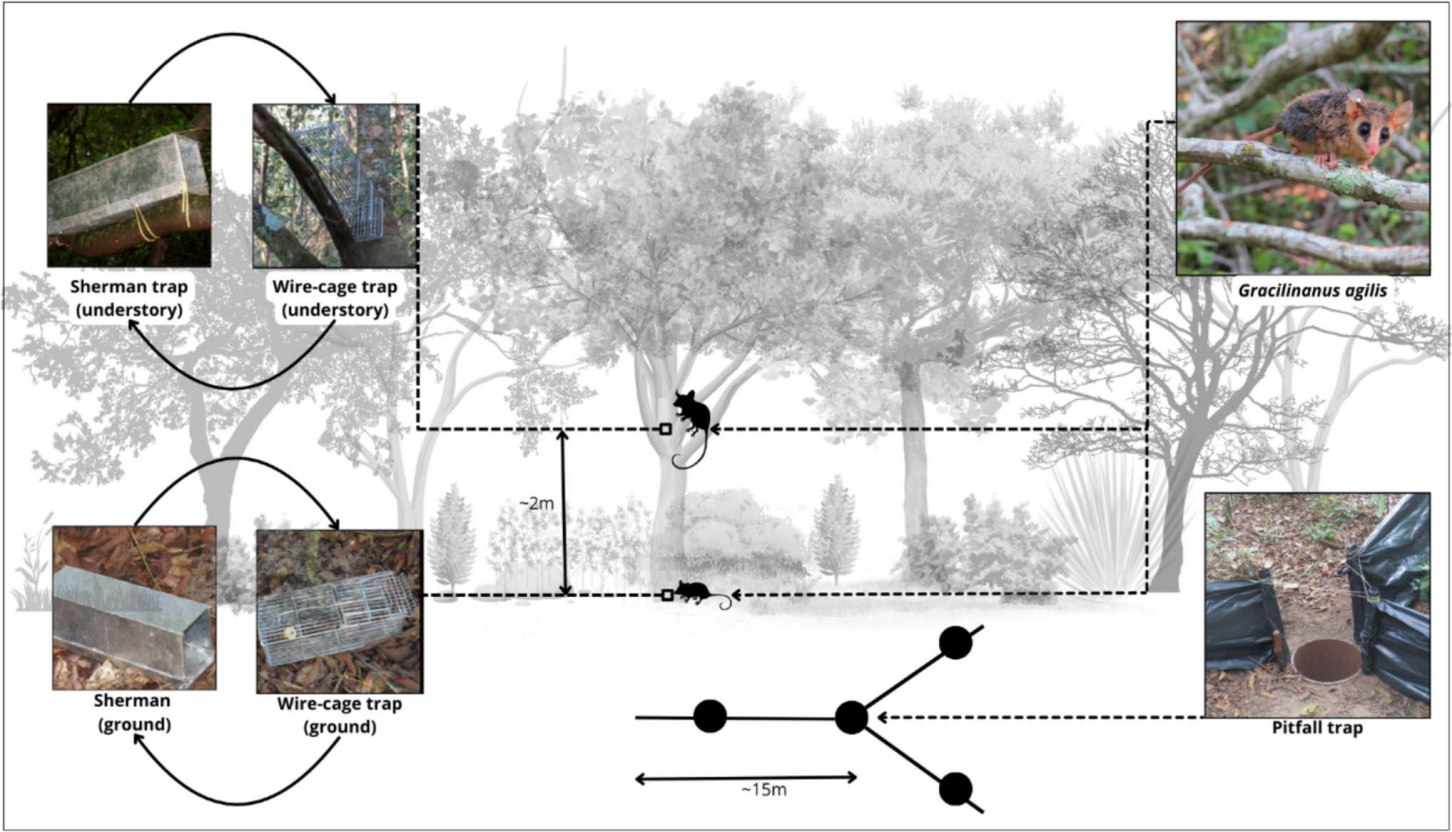
Trap type and positions used to capture *Gracilinanus agilis* in the fragmented landscape of Serra da Fortaleza Wildlife Refuge and its surrounding in southern Goiás, central Brazil

To survey small mammals in the fragmented landscape, we conducted campaigns every 3 months, from January 2020 to December 2023. Five hexagons were sampled per campaign, covering both dry and rainy seasons, totaling 20 hexagons by the end of the year. We captured *G. agilis* using wire-cage traps (30 × 13 × 13 cm) and Sherman traps (25 × 8 × 9 cm) distributed across ten capture stations along two 60-m transects (five capture stations per transect), spaced 15 m apart. Each capture station had two traps (one wire-cage and one Sherman) placed alternatively on the ground and in the understory (1.5 to 2 m high). Additionally, a pitfall trap was set up in each hexagon, consisting of four 60-L buckets arranged in a “Y” shape and connected by a plastic fence (0.8 m high) (Fig. 2). The traps were left open for five to seven nights, totaling 10,400 trap-nights and 1680 bucket-nights.

### Collect of buccal mucosa to micronucleus test

All captured individuals were carefully placed in cloth bags for processing. They were weighed, measured, and had their age determined. Lactating females were given priority in the processing. The buccal mucosa of each animal was scraped using a flexible cotton swab soaked in a drop of physiological solution (0.9% NaCl). The swab was gently rubbed on the right and left buccal mucosa and the roof of the mouth, following Benvindo-Souza et al. (2019). It was then smeared onto clean glass slides (four per individual). After drying, the slides were fixed in a methanol solution for approximately 10 min and stored in a slide holder. The swabs were discarded after use on each animal to prevent contamination of the slides.

The collection and handling of specimens were conducted with authorization from the Instituto Chico Mendes de Conservação da Biodiversidade (ICMBio, permits no. 69328-1, 69328-2, 79453-1) and approved by the Ethics Committee on the Use of Animals at the State University of Goiás (CEUA-UEG, no. 009/2019).

In the laboratory, the slides with the samples were stained with a Rapid Dye kit (Panoptic/Laborclin). To remove the excess staining solution, we rinsed the slides with distilled water. We counted 1000 cells per subject under a Leica microscope at ×100 magnification. Additionally, we use the relative frequency to provide a standardized index (Carrard et al. 2007), calculated as the number of cells with nuclear abnormalities divided by the total number of cells analyzed.

The following nuclear abnormalities were evaluated (following Bolognesi et al. 2013; Benvindo-Souza et al. 2019): micronuclei, karyolysis, pyknosis, nuclear bud, binucleated, pyknosis, karyorrhexis, and karyolysis.

### Environmental variables

To assess environmental quality, we measured various habitat and landscape characteristics within each hexagon, including vegetation structure, resource availability, forest cover and density, distance to water, distance to forest edges, and soil chemistry.

To evaluate vegetation structure and resource availability, we randomly selected five capture stations within each hexagon and established a 5-m radius around them. Within this area, we counted the number of trees (woody plants with DBH > 15 cm), shrubs, fallen logs, and lianas. Additionally, we measured canopy and litter cover at five points: one central point near the traps, and four additional points in the cardinal directions, each 5 m away (see Freitas et al. 2002).

Resource availability was assessed by collecting litter within 0.50 × 0.50 m quadrat and collecting arthropods in pitfall traps (500-ml cups containing water and detergent) for approximately 48 h. The collected materials were screened in the laboratory, where plant biomass (fruits) and animal biomass (arthropods) were weighed.

Forest cover was determined by calculating the proportion of forested area within each hexagon using the raster “brasil_sentinel_coverage_2022.tif” (https://brasil.mapbiomas.org/). Forest density was assessed using the Normalized Difference Vegetation Index (NDVI), obtained from satellite images (http://earthexplorer.usgs.gov/) using bands 4 (Red) and 5 (near infrared). NDVI was then calculated using the formula: NDVI = (Near-infrared − Red)/(Near-infrared + Red). We measured the distance from the centroid of each capture station to the nearest watercourse and forest edge. These measurements were obtained using Quantum GIS v.3.16.3 program (QGIS 2022).

To characterize soil chemical properties, we collected five samples along the capture station in each hexagon. Approximately 500 g of soil was collected from each point at a depth of up 20 cm, stored in plastic bags, and kept at room temperature (Filizola et al. 2006). The samples were then analyzed in a specialized laboratory to determine their chemical composition.

### Statistical analysis

To compare the differences in the frequency of anomalies between the types of nuclear abnormality, between individuals at the edge and inside of the fragments and the interaction between these factors, we used the Alignment and Rank Transformation (ART) test through the “ARTool” package (Kay et al. 2021). The ART test involves aligning the data for each effect and then applying a rank transformation. After the transformation, a standard Analysis of Variance—ANOVA is applied to the transformed data. This approach was used because the data did not meet the assumptions of normality and homogeneity.

To investigate the association between the frequency of nuclear abnormalities (response variable) and vegetation structure, resource availability, landscape metrics, and soil variables (explanatory variables), we used multivariate analysis. First, we reduced the multicollinearity between the predictor variables within each matrix (vegetation structure, resource availability, and landscape metrics [habitat structure] and chemistry composition [soil variables]) by means of a Pearson correlation analysis, considering associations with a coefficient greater than 50% (R >|0.5|) to be statistically significant. We then compared the association between the matrices by means of a Mantel correlation test, using the “mantel” function from the “vegan” package (Oksanen et al. 2025). In addition, we used a Canonical Redundancy Analysis (RDA) to explore the relationship between a set of nuclear abnormalities (matrix Y) and a set of explanatory variables, previously selected by the Mantel test matrix association (habitat structure matrix, soil matrix, or both). We used residual analysis to verify the assumption of linearity between the matrices. We also used the permutation of the response matrix values to assess the significance of the relationship between the response and explanatory variables, using anova.cca() with 999 permutations to test the significance of the terms in the model and of each canonical axis.

## Results

**Fig. 3:**
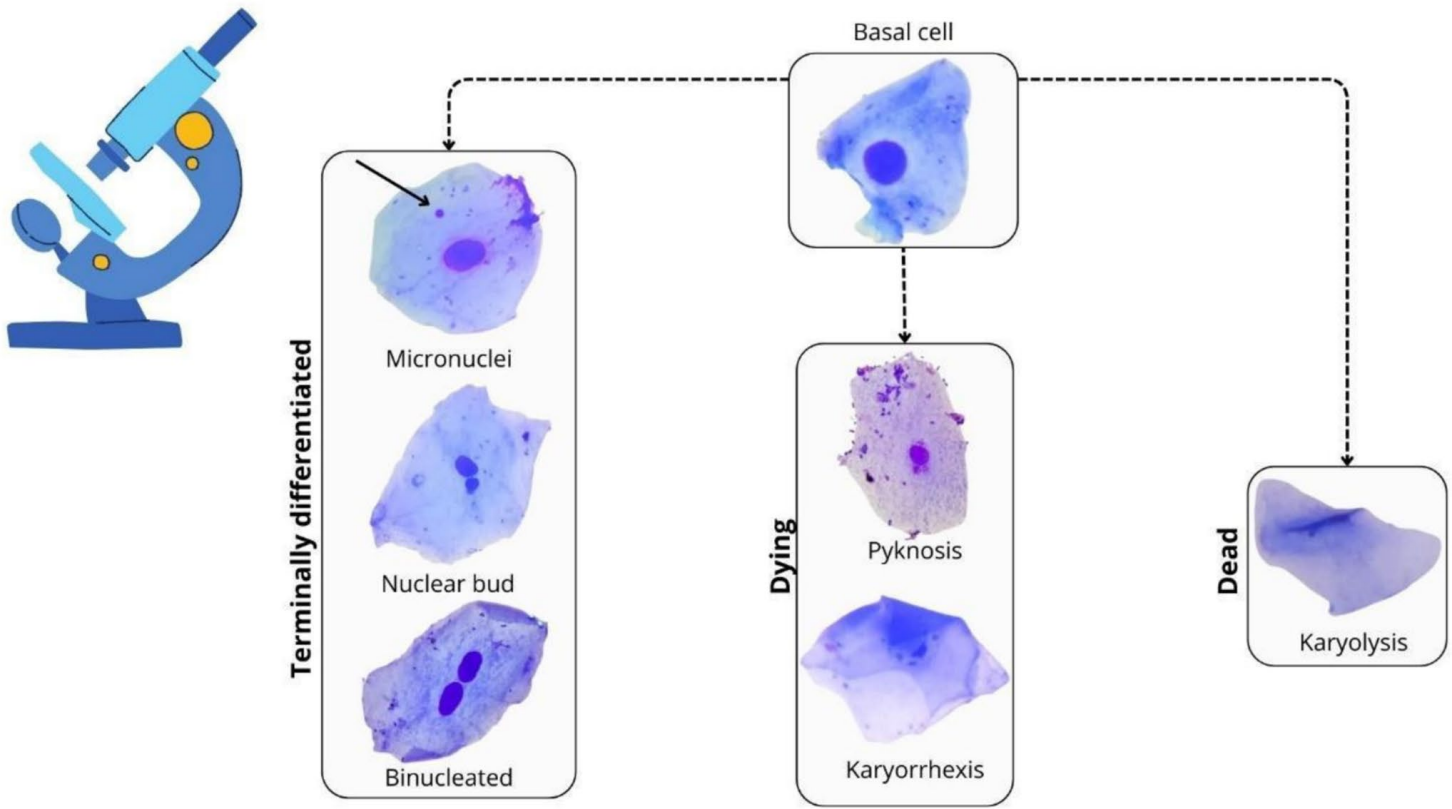
Photomicrograph of different types of abnormalities in exfoliated buccal mucosa cells of *Gracilinanus agilis* in a fragmented landscape in central Brazil. Magnified ×1000

We captured *G. agilis* in 13 hexagons (Fig. 1), obtaining 41 individuals (13 ♀; 28 ♂; an average of three per hexagon). Micronuclei, nuclear bud, binucleated cells, pyknosis, karyorrhexis, and karyolysis were the types of nuclear abnormalities found in exfoliated cells of the buccal mucosa of *G. agilis* (Fig. 3).

**Fig. 4:**
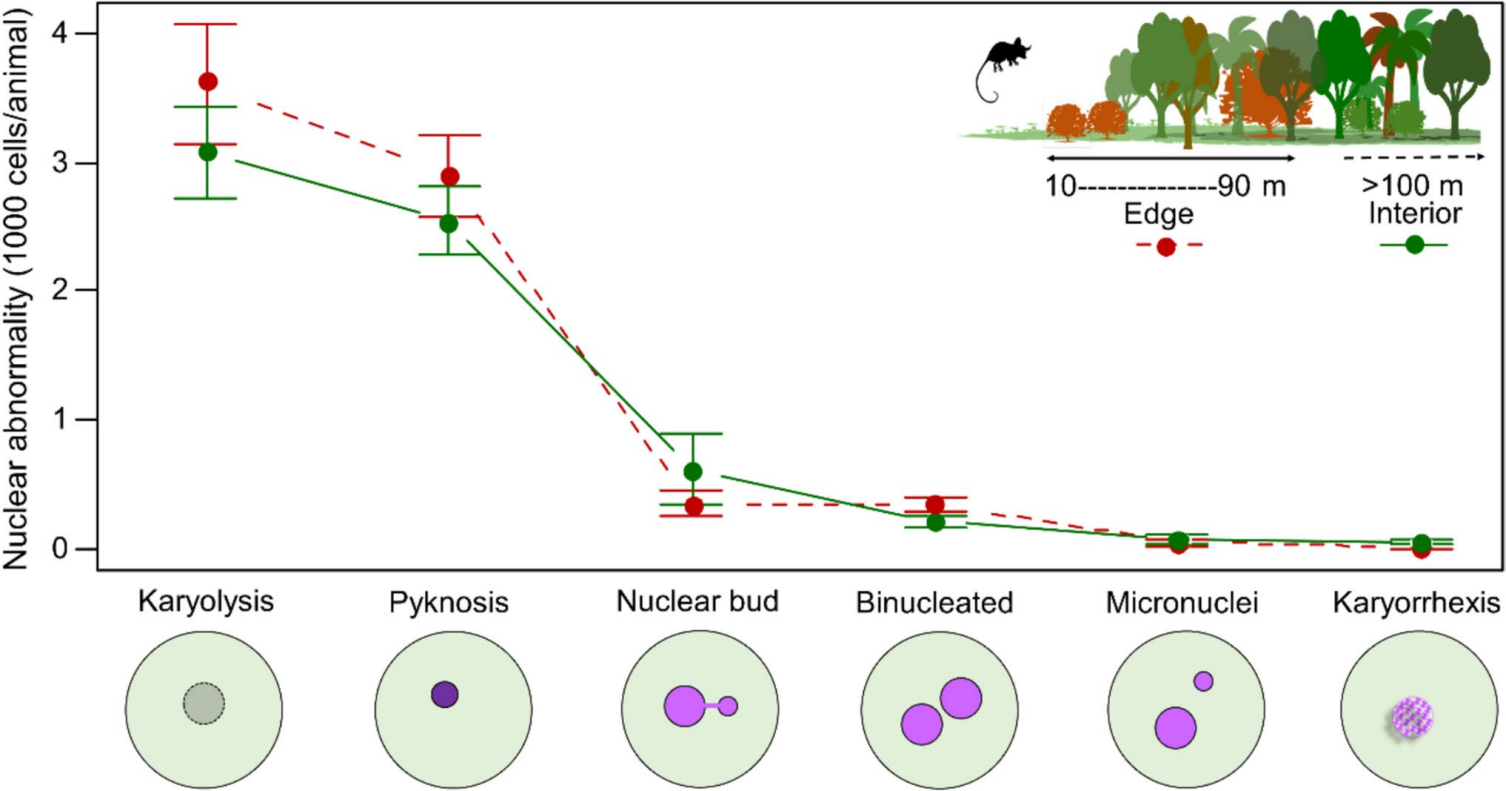
Mean and standard deviation of the frequency of different types of nuclear abnormalities in *Gracilinanus agilis* captured at the forest edge and in the interior of the semideciduous fragments in central Brazil

Karyolysis (mean = 3.32 ± 1.81 standard deviation) and pyknosis (2.70 ± 1.31) were the most frequent abnormalities in the exfoliated buccal mucosa cells of the marsupial *G. agilis*, compared to other types of abnormalities (*F* = 119.25, df = 5, *p* < 0.001). We also found a significant difference (*F* = 9.98, df = 1, *p* = 0.002) in the frequency of nuclear abnormalities between individuals captured at the forest edge and those from the interior of the fragments. The mean frequency of karyolysis (3.61 ± 2.01), pyknosis (2.89 ± 1.40), and binucleated cells (0.34 ± 0.24) was higher in individuals captured at the forest edge compared to those from the interior (karyolysis = 3.08 ± 1.64; pyknosis = 2.54 ± 1.24; binucleated = 0.21 ± 0.21) (Fig. 4). On the other hand, we found no significant interaction (*F* = 2.06, df = 5, *p* = 0.07) between abnormality types and edge effect.

**Fig. 5:**
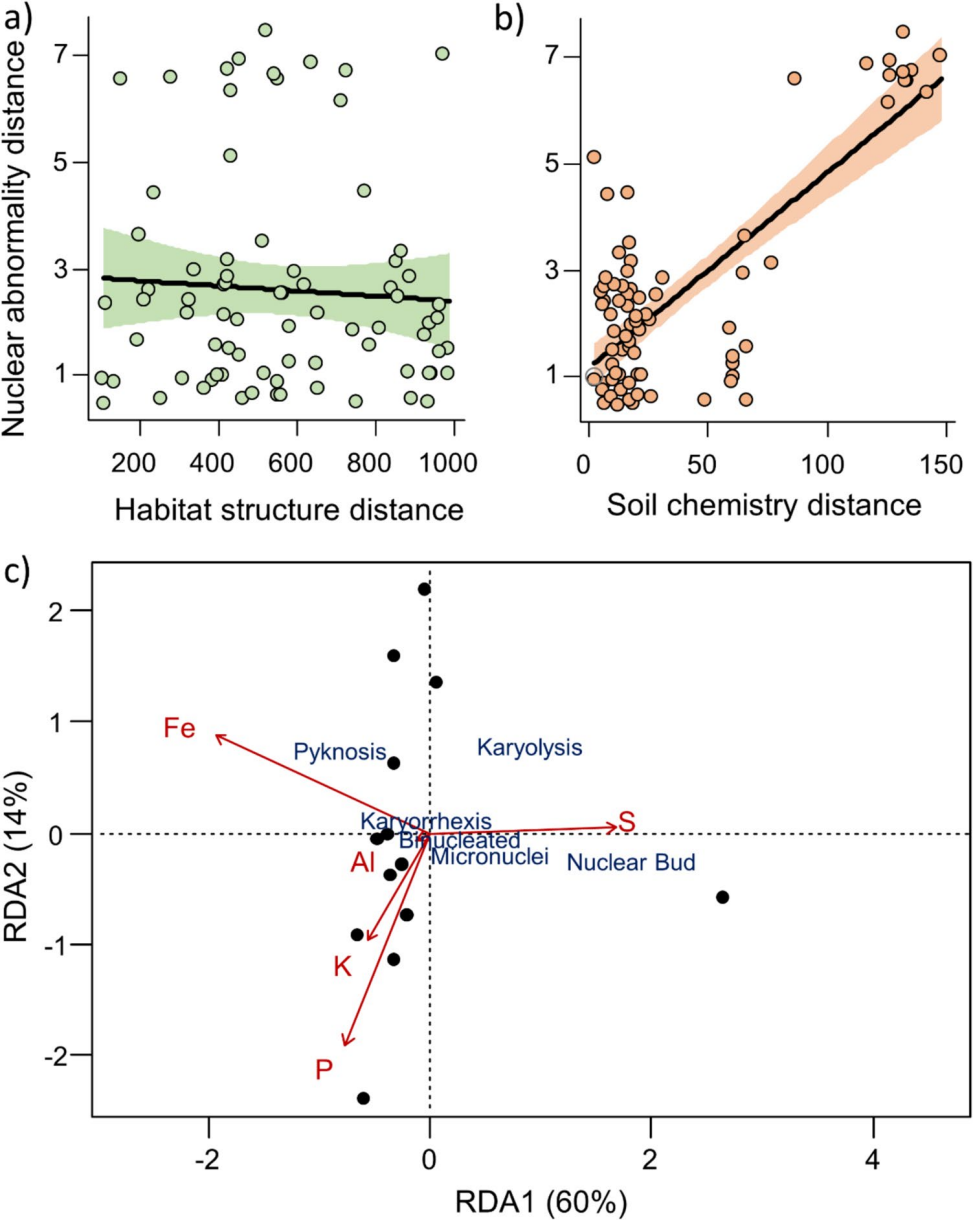
Mantel test correlations between pairwise distances in nuclear abnormalities and (**a**) habitat structure and (**b**) soil chemistry. **(c)** RDA biplot showing the association between soil chemical elements and the frequency of nuclear abnormalities in *Gracilinanus agilis* within a fragmented landscape in central Brazil

We found no association between the distance matrices of nuclear abnormalities and habitat structure (Mantel: *r* = −0.061, 0.491). On the other hand, the distance matrix of the nuclear anomaly variables was associated with the matrix of soil chemistry variables (Mantel: *r* = 0.78, *p* = 0.03). For this association of matrices, the first two axes of the RDA explained 74% of the variation, with the model being statistically significant (*F* = 4.382, df = 5, *p* = 0.034). The first axis accounted for 60% of the variation, with good explanatory significance (*F* = 17.461, df = 1, *p* = 0.026). The sulfur was statistically (*F* = 9.632, df = 1, *p* = 0.036) positioned in the positive portion of axis 1, associated with “nuclear bud” cells (Fig. 5), while iron was statistically (*F* = 6.200, df = 1, *p* = 0.015) positioned in the negative portion of axis 1, associated with “pyknotic” cells (Fig. 5). The phosphorus was statistically significant (*F* = 4.011, df = 1. *p* = 0.011) in the negative portion of axis 2, however, in a position not close to any of the nuclear abnormalities. Aluminum and potassium were not statistically associated with any axis position.

## Discussion

### Micronucleus test

To the best of our knowledge, this is the first in situ study to assess the impacts of genotoxic damage in the South American marsupial *Gracilinanus agilis*, using exfoliated buccal mucosa cells in a fragmented landscape that includes a protected area surrounded by agriculture and pasture matrices. The primary advantage of the micronucleus test is its simplicity, as it requires only exfoliated buccal mucosa cells (Benvindo-Souza et al. 2019). Unlike other techniques that rely on blood or bone marrow samples, the buccal mucosa micronucleus test is a minimally invasive, rapid, and cost-effective method that provides reliable insights into chemical contamination (Thomas et al. 2009; Benvindo et al. 2019).

We identified six types of nuclear abnormalities in the exfoliated buccal mucosa cells of *G. agilis.* Various forms of DNA damage have been documented using this approach (Carrard et al. 2007; Thomas et al. 2009; Bolognesi et al. 2013; Benvindo-Souza et al. 2019). The presence of micronuclei, nuclear buds, and binucleated cells represents an early stage of cellular damage, ultimately leading to terminal differentiation. This damage results from DNA injuries (micronuclei and nuclear buds) and defects in cytokinesis (binucleated cells). In contrast, the appearance of others nuclear abnormalities, such as karyolysis, pyknosis, and karyorrhexis, is indicative of cell death (Carrard et al. 2007; Thomas et al. 2009; Bolognesi et al. 2013).

Both, karyolysis and pyknosis, is a reflection of different stages of nuclear degeneration, often resulting from severe genotoxic damage. Karyolysis is characterized by the progressive dissolution of the cell nucleus due to chromatin degradation. Primarily, this phenomenon occurs in necrotic cells and indicates the loss of genetic material. The process leads to the complete disintegration of the nucleus, making an advanced stage of cell death (Thomas et al. 2009; Bolognesi et al. 2013).

In contrast, pyknosis represents an early stage of nuclear degradation, characterized by chromatin condensation and nuclear shrinkage. Unlike karyolysis, where nuclear material is lost, pyknosis results in a dark, dense, and often spherical nucleus (Thomas et al. 2009; Bolognesi et al. 2013).

While the presence of pyknosis may indicate the early onset of apoptosis or cellular stress responses, karyolysis suggests irreversible damage leading to necrotic death. Thus, the analysis of these nuclear abnormalities, along with others such as karyorrhexis, binucleated cells, nuclear bud, and micronucleus formation, provides a robust approach for evaluating genetic and cytotoxic damage in different species (Carrard et al. 2007; Thomas et al. 2009; Bolognesi et al. 2013), reinforce that these cellular abnormalities serve as reliable biomarkers for assessing genotoxicity in marsupials like the Agile Gracile Opossum.

### Edge effect on DNA damage

We found a higher frequency of cellular abnormalities, particularly karyolysis and pyknosis, in the exfoliated buccal mucosa of *G. agilis* captured at the forest edge compared to the interior of the fragments. These abnormalities are associated with cell death (Bolognesi et al. 2013). The edge effect on DNA damage has been reported in bats exposed to urban pollution near highway bridges, where high frequencies of micronuclei and other nuclear abnormalities were linked to the ingestion of contaminated prey from cities or nearby farmland (Benvindo-Souza et al. 2019). Similarly, we believe that the increased frequency of nuclear abnormalities in *G. agilis* at the forest edge could result from greater exposure to contaminated prey, particularly with heavy metals and pesticides from surroundings corn, soybeans, and pasture fields. *Gracilinanus agilis* feeds on an important hemipteran pest of crops (Camargo et al. 2022) and the widespread, often unregulated, use of pesticides in these plantations (Corguinha et al. 2015; Skidmore et al. 2023) may contribute to genotoxic stress. Thus, proximity to anthropogenic activities may increase genotoxic risk in wildlife.

Food and water consumption are the primary route of heavy metal contamination (Amaral et al. 2012; Zukal et al. 2015), followed by dermal absorption and inhalation (Ferrante et al. 2018; Buchweitz et al. 2018). Insectivorous species are particularly vulnerable, as numerous studies have shown that insects can bioaccumulate both insecticides and heavy metals, making them a significant source of contamination (Stansley et al. 2001; Hernout et al. 2016). In this context, *G. agilis* is especially susceptible to pesticide exposure due to its role as a natural predator of agricultural pests (Camargo et al. 2022). This concern is further supported by the presence of various arthropods in the fecal samples of *G. agilis* (Claro and Hannibal 2022). However, further research is needed to confirm the extent of contamination through this pathway.

### Habitat and soil chemistry on DNA damage

We found no association between habitat structure and the frequency of nuclear abnormalities. However, soil chemistry was linked to the frequency of DNA damage, with sulfur (S) and iron (Fe) especially associated with the occurrence of nuclear buds and pyknotic cells, respectively.

The Cerrado is one of Brazil’s main biomes for grain and livestock production. As of 2023, 47.21% of its original area was occupied by agriculture and livestock farming (brasil.mapbiomas.org). Due to its proximity to agricultural areas, pesticide particles and other xenobiotic substances may have altered the soil’s chemical composition. Monocultures such as soybeans, corn, and sugarcane rank among the highest consumers of pesticides in Brazil (Pignati et al. 2017), and the state of Goiás is consistently among the top agrochemical users nationwide (IBAMA 2024). The proximity between native habitats and croplands therefore increases the likelihood of soil, water, and biota contamination by these substances.

Studies conducted in agricultural areas commonly associated with soybean and corn cultivation have detected the presence of heavy metals such as arsenic (As), cadmium (Cd), lead (Pb), iron (Fe), and maganese (Mn), as well as pesticide residues including glyphosate, atrazine, and organophosphates (Corguinha et al. 2015; Dias et al. 2023; Skidmore et al. 2023). These contaminants are known to promote oxidative stress, disrupt enzymatic antioxidant defenses, and interfere with DNA repair and replication, ultimately leading to chromatin condensation, nuclear fragmentation, and cell death (Bolognesi et al. 2013; Ferrante et al. 2018). In this context, the chemical profile of the soil likely reflects a mixture of agricultural inputs capable of eliciting genotoxic and cytotoxic effects in resident organisms.

In the case of *G. agilis*, exposure to heavy metals and pesticide residues from the surrounding matrix could be a key factor contributing to cytogenetic damage observed in individuals from the forest edge. This hypothesis aligns with the species’ insectivorous diet and small home range, which increase the likelihood of trophic and environmental exposure (Shibuya et al. 2017; Camargo et al. 2022). Edge environments are typically more exposed to agricultural runoff and aerial drift of agrochemicals, which could explain the higher frequency of nuclear abnormalities detected in individuals from the areas. Thus, our findings suggest that chronic exposure to agrochemical-derived contaminants, mediated by both soil contact and diet, contributes to the genotoxic patterns observed in the Agile Gracile Opossum.

Despite the presence of nuclear abnormalities, further studies using the micronucleus test on *G. agilis* are needed to confirm its effectiveness in wildlife assessments. While our findings advance the understanding of soil chemical elements in the nuclear abnormalities of the buccal mucosa of the Agile Gracile Opossum, significant knowledge gaps remain. Thus, integrating additional biomarkers for genotoxicity and mutagenicity, similar to the approach used in rodent ecotoxicology (Claro et al. 2024), could enhance the elevation of environmental contamination in marsupials. Future research should focus on identifying specific soil and water pollutants and exploring additional non-invasive biomarkers. Moreover, further investigation to the food chain is essential to determine whether *G agilis*’ diet is contaminated and to clarify the relationship between this contamination and the frequency of nuclear abnormalities.

## Conclusion

This study represents the first in situ assessment of genotoxic impacts on the South American marsupial *Gracilinanus agilis*, utilizing exfoliated buccal mucosa cells in a fragmented landscape surrounded by agricultural and pasture matrices. The application of the micronucleus test, a non-invasive biomarker, proved effective in detecting nuclear abnormalities, reinforcing its utility for wildlife biomonitoring.

Our findings identified six types of nuclear abnormalities in *G. agilis*, with karyolysis and pyknosis being the most frequent. Individuals captured at the forest edge exhibited significantly higher frequencies of nuclear abnormalities compared to those from the fragment interior, suggesting that proximity to anthropogenic activities increases genotoxic stress. Given that *G. agilis* is an insectivorous species that preys on agricultural pests, its exposure to heavy metals and pesticides from surrounding corn, soybean, and pasture fields likely contributes to the observed cytogenetic alterations. Soil chemistry, particularly sulfur and iron concentrations, was significantly associated with specific nuclear abnormalities. The widespread use of agrochemicals in this region may be altering soil composition, contributing to the bioaccumulation of toxic substances in local fauna.

Although this study advances the understanding of genotoxic impacts on Neotropical marsupials, further research is needed to strengthen these findings. Future studies should incorporate additional biomarkers for genotoxicity and mutagenicity, as applied in rodent ecotoxicology, to enhance the evaluation of environmental contamination in marsupials. Moreover, detailed investigations into pollutants present in soil and water, along with an assessment of the food chain, are necessary to clarify the link between dietary exposure and nuclear abnormalities in *G. agilis*. Establishing the species’ sensitivity and quantifiable responses to specific environmental stressors will be essential to confirm its potential as a biomonitoring organism. Such efforts will be crucial in refining conservation strategies and mitigating the effects of anthropogenic activities on wildlife health.

## Data Availability

Data available on request from the authors.
